# Le diabète sucré de type I chez l’enfant de moins de 5 ans: à propos d’une observation aux cliniques universitaires de Lubumbashi et revue de la littérature

**DOI:** 10.11604/pamj.2017.26.170.11876

**Published:** 2017-03-24

**Authors:** Maguy Ngongo Omoy, Didier Munganga Ngoy, Eric Kasamba Ilunga, DonDieu Bafwafwa Ntumba, Gray Kanteng a Wakamb, Stanis Wembonyama Okitosho, Oscar Luboya Numbi

**Affiliations:** 1 Université de Lubumbashi, Faculté de Médecine, Département de Pédiatrie, Cliniques Universitaires de Lubumbashi, République Démocratique du Congo; 2 Université de Lubumbashi, Faculté de Médecine, Département des Sciences de Base, Laboratoire des Cliniques Universitaires de Lubumbashi, République Démocratique du Congo

**Keywords:** Ngongo Omoy Maguy, Université de Lubumbashi, Faculté de Médecine, Département de Pédiatrie, Cliniques Universitaires de Lubumbashi, République Démocratique du Congo, T1DM, child, Africa, Lubumbashi

## Abstract

Le diabète sucré de type I (DT1) est en évolution dans le monde, affecte de plus en plus les enfants de moins de 5ans. Sa prise en charge est très délicate plus l’enfant est petit. Si non diagnostiqué à temps ou mal pris en charge, le décès arrive rapidement de suite des complications aigues sévères. Il y a lieu d’être regardant, de sensibiliser la population et d’adapter nos systèmes de santé, face à cette nouvelle épidémie des maladies non transmissibles en Afrique, d’en créer des registres pour en étudier les caractéristiques épidémiologiques dans nos milieux. L’objectif était de montrer l’évidence et la gravité de la survenue de plus en plus précoce de l’âge de diagnostic du DT1 de l’enfant dans notre milieu.

## Abstract (en)

Worldwide trends in type I diabetes mellitus (T1DM) are changing. It is increasingly affecting children of less than 5 years. The smaller the child, the greater the complexity of patient management. A delay in diagnosis or poor treatment can cause sudden death due to acute severe complications. It would be worth looking at this, raising public awareness, adapting our healthcare system to face this epidemic of noncommunicable diseases in Africa and establishing registers to analyze the epidemiological characteristics of type I diabetes mellitus in our environment. The aim of our study was to highlight the seriousness of type I diabetes mellitus occurrence at an early stage in children in our environment.

## Introduction

L’incidence du DT1 est en augmentation dans le monde: 3% par année, accroissement rapide attribué au changement des facteurs environnementaux [1, 2]. Plus de 79.100 enfants ont fait le DT1 dans le monde en 2013, 80 % des personnes atteintes de diabète en général vivent dans les pays à faible et moyen revenu [2, 3]. Le DT1 déclaré chez l’enfant est associé à une surmortalité, ce sont les plus jeunes enfants qui en sont les plus à risque, parce que sa prise en charge très spécifique, relève de la sur spécialité [1, 4, 5]. Pour les pays où le système de santé est à ressources limitées, le problème est plus grave, souvent le DT1 n’est pas diagnostiqué chez l’enfant en Afrique, s’il l’est, le décès arrive prématurément [5, 6]. En République Démocratique du Congo (RDC), comme dans la plupart des pays de l’Afrique sub-saharienne, les données relatives à l’incidence du DT1 de l’enfant sont rares ou inexistantes [2, 4, 7]. L’objectif était de montrer l’évidence et la gravité de la survenue de plus en plus précoce de l’âge de diagnostic du DT1 de l’enfant dans notre milieu.

## Patient et observation

Il s’agissait d’un petit enfant de sexe masculin, âgé de 3ans, pesant 17 Kilos, reçu aux cliniques universitaires de Lubumbashi pour fièvre, rhume, toux,anorexie et douleurs abdominales. Sa température était à 38,8°C, ses amygdales rouges et tuméfiées. Une rhino-amygdalite avait été retenue et prise en charge comme telle. Le lendemain, le patient fut ramené pour vomissements, polyurie, soif et persistance des premiers symptômes. Il était déshydraté, polypneique, somnolant. Le même diagnostic était reconduit; le paludisme et une infection urinaire étaient à exclure. Dans le bilanpara clinique réalisé d’urgence, la glycémie à 586mg% va conduire à rechercher la glycosurie revenue à 4 croix, la cétonurie à 3 croix, l’albuminurie à 2 croix et le pH urinaire à 6, sans leucocyturie. La goutte épaisse était négative. C’est alors que le diagnostic d’une acidocétose diabétique fut retenu; une réhydratation au sérum physiologique et une insulinothérapie intensive à la pompe instaurées. Un relais en sous cutané entrepris par après. A noter que le patient avait été traité 2 semaines avant pour paludisme et pharyngite; que la notion de polyurie avait était signalée mais banalisée. A la deuxième consultation cette notion n’a été ni évoquée, ni recherchée, pendant qu’elle était toujours là. Les antécédents par rapport au diabète dans sa famille n’avaient pas été retrouvés. Pour le reste des examens, L’hémoglobine glyquée 6 jours après le traitement intensif était à 6,9%; l’anticorps antiacide glutamique décarboxylase(GAD65) isolé à 46UI/ml (valeurs normales <30UI/ml) ainsi que l’anticorps anti tyrosine phosphatase (IA2) à 3,26UI/ml (valeurs normales < 7,5UI/ml). La C-peptide n’a pu être dosée. L’écouvillonnage de la gorge avait isolé le streptococcus sensible à la cefotaxime, à la norfloxacine et à l’ Imipenème. Au vu de tous ces éléments, le diagnostic de diabète auto-immun de type 1 a été confirmé, compliqué d’une acidocétose sur un fond d’infection et l’antibiothérapie adaptée.

## Discussion

L’élévation de l’incidence du DT1 de l’enfant serait due non à l’augmentation globale des nouveaux cas, mais plus à un décalage vers un plus jeune âge du début de la maladie, notamment vers les enfants âgés de moins de 5 ans, traduction des facteurs environnementaux trop propices [1]. Notre patient avait 3 ans. Le paradoxe en Afrique réside sur un écart entre une urbanisation galopante à la base de modification des modes de vie, de l’épidémie des maladies non transmissibles dont le diabète et les systèmes de santé qui restent à ressources limitées [6, 7]. Le DT1 chez les tout-petits enfants comporte des particularités, les symptômes manquent de spécificité, sont d’une grande banalité et peuvent facilement être attribués à d’autres pathologies [1]. Notre patient avait les signes d’une cétose diabétique, ce diagnostic n’avait pas était envisagé d’emblée. A ces problèmes, ajouter les moyens économiques limités des parents, face à un coût prohibitif d’un traitement qui par ailleurs est très contraignant, plus l’enfant est petit; il se pose souvent un problème d’erreur, de retard diagnostic et de difficulté de prise en charge [4, 7]. Ainsi, le DT1 de l’enfant est souvent découvert sous forme d’acidocétose diabétique inaugurale, complication potentiellement mortelle et 90% de décès arrivent prématurément chez les enfants atteints de DT1 en Afrique, en moins d’une année après le diagnostic qui souvent n’est même pas posé [2, 4, 7]. Ça serait la cause d’une prévalence apparemment faible du DT1 de l’enfant en Afrique [2]; de quoi attirer l’attention des cliniciens.

Le DT1 est insulinodépendant, la mise en évidence d’un ou de plus d’un des auto-anticorps confirme le diagnostic [8, 9]. Chez notre patient, l’anticorps antiGAD65 avait été isolé positivement à 46 UI/ml, l’anti IA2 faiblement à 3,26 UI/ml. L’hémoglobine glyquée (Hb A1C) dosée 6 jours après une insulinothérapie intensive à la pompe était à 6,9 %, ce qui ne nous a pas permis de connaitre sa valeur initiale avant ce mode de traitement qui la fait rapidement chuter , exposant à des hypoglycémies iatrogènes [1]. La c-peptide n’a pu être dosée, ce qui ne nous avait pas permis de prédire sur une transition éventuelle par une période de lune de miel chez notre patient, période survenant néanmoins à un taux faible à moins de 5 ans [10]. Face à toutes ces difficultés de diagnostic et de prise en charge du DT1 de l’enfant dans nos milieux, l’idéal serait un dépistage précoce des enfants à risque. Chose controversée dans la littérature [8, 9] et très difficile à réaliser dans nos contextes, car le coût très élevé, test souvent indisponible, aussi l’absence des anticorps et même l’inexistence d’antécédent de diabète dans la famille n’exclut pas la possibilité qu’un enfant puisse développer un jour le DT1. Même si le test s’avérait positif, il n’existe pas de traitement préventif à ce jour, car aucune immunomodulation préventive (immunosuppresseurs, insuline en sous cutané ou oral, nicotinamide…) ne s’est avérée efficace pour empêcher l’apparition du DT1 [8-10].

## Conclusion

Le DT1 de l’enfant est bien présent dans notre milieu, même chez les tout- petits enfants de moins de 5 ans. Sa clinique étant moins précise plus l’enfant est petit, il convient de le rechercher devant toute notion de polyurie voire d’énurésie. L’éducation de la communauté sur la surveillance des signes annonçant le début du diabète, la capacitation de nos systèmes de santé, l’implication étatique, peuvent aider au diagnostic et à une prise en charge précoce et efficace; pour améliorer espérance et qualité de vie des enfants atteints de DT1. L’élaboration des registres pour l’étude des caractéristiques épidémiologiques du DT1 de l’enfant dans notre milieu devient une impérative.
